# Bullous Iodide Rash

**Published:** 1909-09-11

**Authors:** 


### Dermatology.

### BULLOUS IODIDE RASH.

The fact that iodides and bromides may produce very troublesome and extensive skin eruptions is well enough known, but it is perhaps little recognised that these eruptions are not confined solely to a pustular or acnëiform type. The bromide rashes may be extremely puzzling sometimes, unless the thought of a drug rash happens to pass through the observer's mind. The same applies to the iodide rashes, one rare form of which is the bullous type, as in the following case:—

A young woman, aged 30, was under treatment by a non-qualified person, and she had been taking for three days a mixture prescribed by him. She then began to develop painful sensations in the skin of her face, forearms, wrists, and thighs, and then there came a reddish brown eruption of what were at first large papules, and which shortly afterwards developed into bullæ. A chemist was consulted the next day, and he ordered another mixture which the patient said had the same taste as the first. She took a tablespoonful of this, and half an hour afterwards she found her skin eruption to be rapidly spreading with the formation of fresh bullæ, and just previous to the eruption of each one of these there was a great deal of pain accompanied by a sense of burning in that area of the skin upon which the bullæ was about to appear. By that evening she was extensively covered with blebs upon her nose, cheeks, chin, forearms, wrists, the dorsal surfaces of her hands and fingers, and the antero-internal region of her legs. In some places the fluid of the early blebs was beginning to dry up with the formation of crusts, but there was no formation of pus. The features were so distorted and disfigured as to be almost unrecognisable. The least pressure upon the skin produced so much pain that the patient cried out with it. The mixtures the patient had been taking were analysed, and both were found to contain potassium iodide. Apparently both the unqualified man and the chemist had assumed that the patient was syphilitic and ordered the same medicine. The amount of the salt that she had taken was by no means extensive however, averaging apparently about ten grains to the dose.

Bullous iodism of this kind is not to be seen every day, of course, but the case illustrates how very severe it may be, and how rapidly it may come on after the drug has been taken. The treatment consisted simply of stopping the medicine, some pills containing opium and extract of belladonna being prescribed for the relief of pain, the belladonna acting probably as a direct antidote to the iodism according to the teaching of d'Aubert. This is perhaps done by opposing the vaso-dilatation produced by the iodine. The recovery was rapid and complete.

